# “Imagining the world anew”: a transformative, rights-based agenda for UHC and SRHR in 2021 and beyond

**DOI:** 10.1080/26410397.2021.1883805

**Published:** 2021-03-01

**Authors:** Anna Gruending, Pete Chapman, Veloshnee Govender

**Affiliations:** a Consultant, Partnership for Maternal, Newborn & Child Health, World Health Organization, WHO, Geneva, Switzerland; b Managing Editor, Sexual and Reproductive Health Matters, London, UK. *Correspondence*: pete.chapman@srhm.org; c Scientist, Department of Sexual and Reproductive Health and Research, World Health Organization, Geneva, Switzerland

## Introduction

Universal health coverage (UHC)^1^ is a monumental idea that gained prominence in the twentieth century, following earlier historical antecedents. Particularly, since the end of the Second World War, the adoption of measures by some governments to ensure – to varying degrees – needed health services without financial hardship has transformed the health and lives of billions of people.

However, demonstrated through growing evidence, we know that the “universal” in UHC remains aspirational. The World Health Organization (WHO) estimates that at least half of the world’s population still does not have full coverage for essential health services and that 100 million people are pushed into extreme poverty because of healthcare costs.^1^ These figures pre-date the tremendous and expanding impact of COVID-19: a crisis which has put enormous strain on, in many instances, under-resourced, poorly functioning and inequitable health systems in high-, middle- and low-income settings, and which – contrary to the initial characterisation of the virus as a “great leveller” – has significantly amplified health inequalities across and within national boundaries.^2^ We also know that comprehensive sexual and reproductive health (SRH) services have often been excluded from health benefit packages (HBPs), which are a necessary requirement for directing policy attention to and resources towards priority needs and services. This exclusion has frequently been the result of pervasive gender inequality and perceived and real political sensitivity and stigmatisation.

The Sexual and Reproductive Health Matters (SRHM) themed issue “Universal health coverage: sexual and reproductive rights in focus” set out to explore the promises and limitations of UHC for sexual and reproductive health and rights (SRHR), with special attention to rights-based perspectives. The issue opened to submissions in 2019 on the heels of the UN High-Level Meeting on UHC.^3^ Despite the sadly familiar diplomatic wrangling around language on SRHR, optimism was generally high as Heads of State adopted what may be the most ambitious global health declaration in history.

However, commitments to UHC – and to SRHR – were immediately put to the test with the onset of the COVID-19 pandemic. By the time that this issue launched in June 2020, the pandemic had already claimed over 400,000 lives, fundamentally altering the global health landscape and priorities, against the backdrop of the impending high-stakes US presidential election and that country’s role as the largest funder of global health and family planning programmes worldwide. The pandemic and the election have served as solemn reminders of how progress towards UHC and SRHR is in lockstep with political agendas and how there remains much work to be done.

As this issue on UHC and SRHR is finalised, we begin a new year with cautious optimism. COVID-19 continues to impact the lives and livelihoods of billions of people, but there is a renewed sense of hope with the continued search for safe, effective and accessible therapeutics and diagnostics,^4^ the approval of several COVID-19 vaccines and the launch of monumental vaccination campaigns,^5^ not to mention the incoming US administration’s commitment to restoring trust in evidence and science,^6^ as well as improved prospects for the financing of global health leadership and of SRH services worldwide.^7^

In this editorial, we reflect on some of the notable findings from this themed issue, and on the prospects for UHC and SRHR in the midst of this transformational moment as countries begin the journey of emerging from the greatest health crisis in over a century.

## Key findings and messages from the issue

Looking back at the call for papers allows us to take stock of ways in which the themed issue, the largest in SRHM (previously RHM)'s 28-year history, has helped to advance the evidence base on SRHR and UHC, and to identify topics that remain under-considered.

### HBP development: the importance of process and participation

One of the objectives of the themed issue was to investigate the extent to which SRHR is comprehensively integrated within national health policies and strategies in settings committed to attaining UHC. This series has arguably been successful in soliciting research on this topic and advancing the evidence base.

The inclusion of comprehensive SRH services within an essential health service package (EHSP) or HBP, particularly in settings where there is fragmentation in funding sources and/or service delivery implementers, represents one of the most important statements of commitment by government towards ensuring equitable access to and delivery of a full package of essential services. However, as increasingly noted elsewhere and within this issue, whilst maternal health, contraception and HIV/AIDS and sexually transmitted infection (STI) services are often – as they should be – included in EHSPs, more contested services (i.e. gender-based violence [GBV], safe abortion and post-abortion care, comprehensive sexuality education and infertility services) are frequently excluded. The extent of their exclusion is very much dependent on prevailing legal and social environments.^8,9^

The constraints imposed by restrictive legal and social environments may be mitigated, and comprehensive SRH services included in EHSPs or HBPs, as Pillay et al. recommend, through *“ … fair and transparent priority setting process[es] [which] consider the best available evidence, burden of disease, and cost effectiveness … underpinned by ethical values such as equity, gender equality and right to health*”.^8^ However, this integral recommendation is challenging to put into practice. Successful follow-through first and foremost requires political will and greater investment in strengthening institutions and capacities of those voices who are often sidelined in policy and technical fora.

The World Health Organization (WHO) recently launched the *UHC Compendium*^10^ to assist national and subnational decision-makers in accessing relevant information towards developing their health intervention packages and related planning and budgeting. In a forthcoming document, WHO will provide specific guidance on the processes and practical steps essential to integrating SRH services into national and subnational health-sector policies and strategies towards UHC. This guidance stresses some critical actions that states can take to provide policy space and accountability mechanisms for the incorporation of SRHR into UHC plans. These include creating deliberative spaces that enable the full participation of all stakeholders in priority-setting processes towards defining EHSPs or HBPs, especially civil society organisations (CSOs) including women’s rights organisations, youth groups and marginalised and vulnerable groups. It also calls for clear criteria for priority setting, transparency on what SRH services are included and reasons for the exclusion of other SRH services, mechanisms for challenging processes if the criteria are found lacking and monitoring the level of financial protection of the SRH services, especially for women and marginalised and vulnerable groups.

### Not losing sight: the challenge of upholding quality while expanding access

Another key lesson that emerges in the themed issue is that, while the inclusion of comprehensive SRH services in HBPs is necessary, and investing in the health system to strengthen its capacity to ensure services are in place to deliver the package is equally critical,^8,11^ these may not be sufficient for ensuring translation of services into accessible and quality services that meet the needs of those at the tail end of society’s pecking order.^12^ If anything, the challenge for quality of care persists even in contexts in which considerable progress towards UHC has been made.

This is especially well highlighted by Juárez-Ramírez et al., who report that access to good quality maternal care (i.e. privacy, freedom of choice, respectful care, availability of translators) from the users’ perspective continues to be a challenge for indigenous women in Mexico.^13^ Appleford et al. propose a “5-P” approach (people, package, provider, payment, polity) to ensuring quality of SRH care within the UHC agenda, arguing for both “systems” and “design” lenses as important steps to quality.^12^ This approach needs to be tested across different settings, particularly in contexts of marked social and income inequalities.

### Emerging country evidence: integrating SRH services into UHC packages

One of the key strengths of the themed issue is a large and diverse number of papers from the country and regional perspectives that allow for sharing of lessons learned and best practices around the integration of SRH into UHC. For instance, the Malaysia case study by Lim et al. identifies key advocacy strategies for the effective prioritisation of SRH services – including GBV services and abortion and post-abortion care – in UHC packages.^14^ Several strategies, including linking SRH issues with international commitments, engaging champions and reframing SRH issues as public health issues, have proven effective to varying degrees in overcoming resistance and elevating these issues in the hierarchy of priorities. The Malaysian case study also highlights the importance of a rights-based approach to SRH and UHC: despite the country’s relative success in reducing maternal mortality over the past decades, the MMR has stalled for the past 15 years at approximately 2.5–3 times its MDG target, largely due to the exclusion of certain high-risk populations, including adolescents, migrants and refugees. This is a stark reminder of the centrality of a rights-based perspective in UHC planning and implementation.

Monga et al. also outline strategies and tactics used to advance the inclusion of SRH services in UHC packages – in this case, safe abortion care in Nepal and Pakistan.^15^ In Nepal, data on the dangers of unsafe abortion were effectively used to reframe the issue as part of the strategy to reduce maternal mortality, and task-shifting from obstetrician-gynaecologists in hospitals to nurse midwives at the community level was encouraged. In Pakistan, CSOs focused their advocacy efforts on influential professional associations and supported the inclusion of safe abortion care in the national midwifery curriculum. One of the key takeaway lessons from both Nepal and Pakistan, echoing findings from elsewhere in the themed issue, is the importance of including local CSOs, especially women’s, youth and professional associations, in UHC processes from the outset, particularly as countries ramp up their efforts to operationalise the UHC Political Declaration.^16^

### Persisting gaps: certain SRH issues and key populations remain on the margins

Just as we find that certain services are typically excluded from HBPs, so too is there a dearth of articles exploring some of these issues and related challenges in greater detail – notably in the areas of GBV, cervical and other gynaecological cancers and sexual function. Perhaps this should not come as a surprise, given that these topics may be considered “newcomers” to a more widely accepted definition of comprehensive SRHR, as put forth by the Guttmacher-Lancet Commission in their 2018 report.^17^ However, a lack of focus on these areas suggests the need to more deliberately seek out evidence on how countries – at various income levels – have successfully integrated these services into their UHC packages.

The call for papers also explicitly sought manuscripts on the rights dimensions of UHC: universalism, equality, non-discrimination and inclusion. On these topics, the series has achieved mixed results. With some notable exceptions, papers exploring barriers and solutions for the provision and uptake of SRH services as part of UHC for marginalised populations are few. For instance, no papers in the issue consider, as a primary aim, the particular barriers faced by LGBTQI populations on accessing SRH services, nor the deeply entrenched challenges that migrants face in utilising health services of many kinds while living on the margins of society and excluded from the very concept of citizenship. The papers that do describe the experiences of marginalised populations remind us of the entrenched inequities that are often perpetuated by existing health systems, and which require sharp focus in UHC reforms if the sexual and reproductive and the wider health and rights of these groups are to benefit.

For instance, Mac-Seing et al. document the continuing challenges that people with disabilities in Northern Uganda face in accessing SRH services, including stigma and discrimination, despite Uganda’s adoption of pro-disability legislation.^18^ As noted earlier, in Mexico, where indigenous women account for a disproportionate number of annual maternal deaths, Juárez-Ramírez et al. highlight both how the standard model of obstetric care continues to fail indigenous women, and the significant gulf between how healthcare personnel and indigenous women themselves explain the barriers to respectful, quality maternal health care.^13^ Both pieces serve as reminders that if the voices and perspectives of populations that continue to face disproportionate marginalisation are excluded, SRH services – even when integrated in UHC systems – are unlikely to have the transformative health impacts to which we aspire.

Consistent with the rising attention to a previously overlooked population group, the issue includes several papers focused on adolescent SRHR. The fundamental requirement for decision-making and agency to be at the forefront of the UHC agenda for adolescent girls and other vulnerable adolescents is highlighted by Ricker and Ashmore in their call to action, as they demand a shift in norms to counteract inequalities and describe opportunities afforded by “glitches” in the gender socialisation process.^19^ Kangaude et al. explore how the framing of laws and policies for adolescent abortion-related care in Ethiopia, Zambia and Malawi, which have varying levels of restriction on access to abortion, is crucial for equity and justice.^20^ Wangamati reiterates the importance of comprehensive sexuality education for adolescents in Sub-Saharan Africa and describes the main challenges to its implementation that impede universal access and the realisation of SRHR.^21^ Though there are clearly enormous gaps to overcome in the prioritisation of adolescent SRHR in national and global SRHR and UHC debates, it is promising to see an increased focus, driven largely by youth-led movements.

### Front and centre: the political lens on SRH and UHC

Finally, a key theme emerging from the issue is that, despite more technocratic aspects of the UHC agenda, UHC and SRH are, by their very nature, nothing if not highly political.

In a commentary highlighting the importance of integrating SRHR within UHC in the context of the pandemic, which has further exposed existing disparities, the former Prime Minister of New Zealand Helen Clark reminds us of the central responsibilities of government in upholding the right to health and argues that strong political leadership is perhaps our most important public health tool.^22^ She calls for greater global cooperation and urges leaders to take the opportunity to “build back better” and protect and strengthen SRHR – services which have been hard hit during the crisis, to the great detriment of women and girls.

In another thought-provoking piece that served as a prelude to the US election, Gilby and Koivusalo decry the politics of silencing SRHR and the potential for its pernicious removal from the UHC agenda on a global scale, with a particular focus on the Trump administration and its “coalition building” in this regard.^23^ Though this global landscape will undoubtedly shift with the incoming US administration, the regressive coalition on SRHR that has grown in size and confidence will likely continue to erect roadblocks in political UHC spaces in the coming years.

Unsurprisingly, politics are also paramount in UHC reforms at country level. While Thailand’s government has been successful in achieving UHC – including SRH – goals, there are still important challenges that must be addressed with regard to SRH services, as outlined by Panichkriangkrai et al. in their commentary.^24^ This piece serves to emphasise that, even when governments are committed to achieving UHC, SRHR requires additional focus. Kabakian-Khasholian et al. describe how, despite a diverse mix of political systems and the impediment of political unrest, the governments of the 11 Arab countries that are considered are making efforts in terms of the integration of SRH services within primary health care systems.^25^ Owino et al. highlight Kenya’s experience in the roll out of UHC via an SRHR lens, concluding that – despite prior optimism – the UHC process under the present government in Kenya falls short in this respect.^26^

## Future perspectives: “righting” the power imbalances in UHC spaces

Reflecting on the richness of this issue and its key themes and messages, we believe that one of the most resonant priorities moving forward is to harness a rights-based approach to reconsider the balance of power in UHC processes. In this regard, we can ask ourselves and the broader UHC and SRHR movements several important questions. First, who holds decision-making power, who has a seat around the table, and who – deliberately or not – has been excluded? Second, how can the SRHR community in general, and women’s groups and more marginalised populations in particular, engage more effectively at country level to influence decisions on the shape, scope and coverage of HBPs? Third (and related to point two), what lessons can be learned from the inspiring civil society mobilisation and engagement on other global health priorities, most notably HIV/AIDS? And finally, how can the UHC and health systems space – which has historically been more technocratic and less rights-centred – be made more inclusive, accessible and rights-focused? Can SRHR, with its inherent focus on rights, accountability and addressing barriers relating to stigma and discrimination, be the propellant that drives change in “righting” the power imbalances in UHC spaces, strengthening trust in health systems and driving collective action towards making UHC a reality?

These questions are as pertinent as ever today in the global health landscape indelibly altered by the COVID-19 pandemic, where trust is severely challenged by, and in some instances has declined because of, pseudoscience, conflicting messages and conspiracy theories. UHC2030’s recent “State of commitment to UHC” report argues that UHC is not a pipe dream that can be put on hold until the pandemic subsides, but rather is part of the solution: increasing investments in UHC and specifically processes that invest in strengthening bottom-up participation can help governments to meet their populations’ needs, to rebuild trust and to “build back better” from the pandemic.^27^ Indeed, many of the world’s great health systems were forged in times of crisis and have since become a fundamental part of the social contract between governments and their citizens.

In a lyrical piece written near the beginning of the pandemic, Indian author Arundhati Roy wrote that historically, “pandemics have forced humans to break with the past and imagine their world anew.”^28^ This moment of great crisis that has brought to the fore the pressing need for stronger health systems and UHC, and the undeniable reality that, when it comes to global health, solidarity is paramount – we will sink or swim together. This, we believe, is the moment to imagine the world anew and to unite around a truly global agenda for UHC – one with the rights, including the sexual and reproductive health and rights, of all people at its centre.
